# Platelet counts modulate the quantitative relationship between hepatitis B viral DNA and surface antigen concentrations: a cross-sectional study of hematological, histological and viral factors

**DOI:** 10.1186/s12879-016-2121-y

**Published:** 2017-01-05

**Authors:** Chao-Wei Hsu, Kung-Hao Liang, Chih-Lang Lin, Tong-Hong Wang, Chau-Ting Yeh

**Affiliations:** 1 Liver Research Center, Chang Gung Memorial Hospital, Linko, Taoyuan city, Taiwan; 2 Chang Gung University, College of Medicine, Taoyuan city, Taiwan; 3 Liver Research Unit, Keelung Chang Gung Memorial Hospital, Keelung, Taiwan; 4 Tissue Bank, Chang Gung Memorial Hospital, Taoyuan city, Taiwan; 5 Research Center for Industry of Human Ecology, Chang Gung University of Science and Technology, Taoyuan city, Taiwan; 6 Graduate Institute of Health Industry Technology, Chang Gung University of Science and Technology, Taoyuan city, Taiwan; 7 Molecular Medicine Research Center, Chang Gung University, Taoyuan city, Taiwan

**Keywords:** Hematology, Hepatitis B natural history, Liver fibrosis, Viral-host interactions

## Abstract

**Background:**

The concentrations of hepatitis B virus (HBV) DNA and surface antigen (HBsAg) are two critical virological variables to be monitored in chronic hepatitis B. HBsAg is derived from the HBV genome. Thus, higher HBV-DNA concentrations should implicate higher HBsAg levels. Nevertheless, the two variables do not manifest a simple linear relationship due to elusive host factor involvements. The aim of this study was to address the discrepancy of HBV DNA and HBsAg levels by a quantitative modeling of HBsAg concentrations.

**Methods:**

Pretreatment hematological, histological and virus serological records of 327 chronic hepatitis B patients were reviewed. Two independent patient cohorts were used for validation.

**Results:**

Univariate/multivariate analysis showed that ISHAK fibrosis stages, HBV-DNA levels and hepatitis e-antigen status were independently associated with HBsAg concentrations. In agreement with the natural history of chronic hepatitis B, HBsAg concentrations were negatively correlated with ISHAK fibrosis stages (adjusted *P* = 0.002). Subgroup analysis showed that significant HBsAg-DNA correlation existed in high-viral-titer patients with HBV-DNA > 6 log_10_ IU/mL (*P* < 0.001), but not in low-viral-titer patients with HBV-DNA ≤ 6 log10 IU/mL (*P* = 0.076). A backward stepwise linear regression analysis in the low-viral-titer subgroup revealed a significant correlation between HBsAg levels and a linear combination of HBV-DNA levels and platelet counts. A biphasic model was thus established to accommodate patients with high and low HBV-DNA titers:

$$ \mathrm{HB}\mathrm{sAg}=0.538\ast \mathrm{H}\mathrm{B}\mathrm{V}{\textstyle \hbox{-}}\mathrm{D}\mathrm{N}\mathrm{A}+0.001\ast \mathrm{platelet}\ast \left(\left|6{\textstyle \hbox{-}}\mathrm{H}\mathrm{B}\mathrm{V}{\textstyle \hbox{-}}\mathrm{D}\mathrm{N}\mathrm{A}\right|+6{\textstyle \hbox{-}}\mathrm{HB}\mathrm{V}\mathrm{D}\mathrm{N}\mathrm{A}\right){\textstyle \hbox{-} }0.321 $$

The estimated HBsAg concentrations correlated well with the measured HBsAg levels not only in the model construction cohort (*N* =327, *P* < 0.001), but also in two validation cohorts comprising respectively the patients who had received pretreatment liver biopsy assessments (*N* = 45, *P* = 0.001), and the treatment-naïve patients who had not received liver biopsy (*N* = 80, *P* < 0.001).

**Conclusion:**

HBsAg concentrations can be quantitatively estimated by viral DNA concentrations and human platelet counts.

## Background

Chronic hepatitis B virus (HBV) infection is an endemic disease with a global burden of 350 million patients [1]. This disease persists for multiple decades, and its natural history comprises the immune tolerance, immune clearance and inactive residual phases [2–6]. During the chronic infection, episodes of liver inflammation may occur which cause progressive liver fibrosis and cirrhosis, leading toward thrombocytopenia [7], hypoalbuminemia [8], portal hypertension, esophageal varices, ascites [9], liver decompensation and hepatocellular carcinoma (HCC) [10]. To prevent such devastating consequences, effective antiviral therapies were now vigorously used, with viral and host status carefully monitored [11]. Serum concentrations of HBV DNA and surface protein antigen (HBsAg) are both important viral markers [12]. HBsAg is derived from the HBV genome. Thus, higher HBV DNA concentrations should implicate higher HBsAg levels.

Despite the established molecular origin, serum HBV DNA and HBsAg did not manifested a simple linear relationship in the natural course. The HBV DNA and hepatitis B e-antigen (HBeAg) levels were drastically reduced in the immune clearance phase, while the HBsAg levels were further reduced continuously in the inactive residual phase [2–6]. The discrepancy between HBV DNA and HBsAg levels made them independent variables rather than confounding variables in clinical studies. For example, they played different roles in the prediction of subsequent HCC [13]. Medical guidelines suggested that anti-viral treatments should be given to patients in the immune clearance phase for the purpose of expediting the natural course into the inactive residual phase; and to patients with viral reactivation during the inactive residual phase [11, 12, 14]. HBV DNA was demonstrated to be effectively suppressed, often to undetectable levels, by treatments of approved nucltos(t)ide analogs including lamivudine [15], adefovir [16], entecavir [17], telbivudine [18] and tenofovir [19]. The HBsAg, however, remained positive for years for most of these treated patients. This was why HBsAg seroconversion (the disappearance of HBsAg and the production of anti-HBs antibody), rather than the HBV DNA undetectability, was regarded as the closest sign of cure [12]. On the other hand, patients with negative HBsAg but with positive HBV DNA were occasionally identified, and referred to as the occult hepatitis B patients [20–22]. These patients were still at risk of HBV reactivation [22].

The lack of linear relationship between HBV DNA and HBsAg may be partly explained by the viral life cycle. The covalently closed circular DNA (cccDNA) is the template for generating messenger RNAs, which are further translated to produce HBsAg, as well as the pregenomic RNAs which are reversely transcribed to viral DNA [23]. Since the HBV DNA can integrate into the human genome, the HBsAg may also be derived from the integrated HBV DNA in addition to cccDNA [24]. The viral life cycles occurred in the human hepatocytes, making them susceptible to host factors.

The discrepancy between serum HBV DNA and HBsAg levels remained to be quantitatively evaluated. Therefore, we employed a data-driven approach and conducted a systematic, multivariate evaluation of hematological, histological and viral factors to evaluate their effects on HBsAg concentrations.

## Methods

### Patients

This study was approved by the institutional review board of the Chang Gung Memorial Hospital, Taiwan, and conducted in accordance with the Declaration of Helsinki. All patients have given informed consent for the deposition of their clinical samples to the tissue bank of Chang Gung Memorial Hospital, Taiwan, for academic researches.

In the first stage, clinical records of 327 chronic hepatitis B patients who received pretreatment hematology, liver histology and viral serology assessments between years 2007–2009 were retrospectively retrieved for a quantitative modeling (Table 1)**.** Liver histology was evaluated by the ISHAK hepatic activity indexes [25]. In the second stage, two independent cohorts were assessed for the validation purposes. The first cohort comprised 45 patients who also received liver biopsy for pretreatment evaluations between years 2007–2009. The second cohort comprised 80 anti-hepatitis B treatment-naïve patients evaluated between years 2010–2012. These patients did not receive liver biopsy.

**Table 1:** Baseline characteristics of patients in the model construction cohort

	Values
Subject number	327
Age	44.21 ± 11.22
Gender	
Male	280 (85.63%)
Female	47 (14.37%)
Liver Histology	
ISHAK Fibrosis Stages	
0	2 (0.61%)
1	40 (12.23%)
2	51 (15.60%)
3	110 (33.64%)
4	33 (10.09%)
5	74 (22.63%)
6	17 (5.20%)
Piecemeal necrosis	
0	101 (30.89%)
1	165 (50.46%)
2	47 (14.37%)
3	14 (4.28%)
Confluent necrosis	
0	307 (93.88%)
1	6 (1.83%)
2	2 (0.61%)
3	1 (0.31%)
4	10 (3.06%)
5	1 (0.31%)
Focal (spotty) lytic necrosis, apoptosis and focal inflammation	
0	2 (0.61%)
1	93 (28.44%)
2	161 (49.24%)
3	67 (20.49%)
4	4 (1.22%)
Portal inflammation	
0	6 (1.83%)
1	77 (23.55%)
2	103 (31.50%)
3	123 (37.61%)
4	17 (5.20%)
5	1 (0.31%)
Viral serology	
HBV DNA (log10 IU/ml)	6.35 ± 1.71
HBeAg positive	146 (44.65%)
HBsAg (log10 IU/ml)	3.33 ± 0.98
Hematology	
ALT (IU/L)	177.31 ± 173.34
AST (IU/L)	106.96 ± 122.57
Bilirubin (mg/dL)	0.98 ± 0.42
Albumin (g/dL)	4.55 ± 0.35
Gamma-glutamyltransferase (IU/L)	59.53 ± 46.75
Platelet (1000/mm^3^)	189.78 ± 50.31
Hemoglobin (g/dL)	15.20 ± 1.36

### Quantitation of HBV DNA and HBsAg concentrations

HBV DNA levels were measured by use of the COBAS AmpliPrep/COBAS TaqMan HBV Test, v2.0 assay (Roche Molecular Systems Inc, Pleasanton, CA) according to manufacturer’s protocols. HBsAg concentrations were measured by use of Elecsys HBsAg II assay (Roche Diagnostics GmbH, Mannheim, Germany).

### Statistical analysis

The HBV DNA and HBsAg concentrations were consistently represented here in the logarithm scale due to their wide numerical ranges. Clinical associations were evaluated by univariate and multivariate linear regressions. Subgroup analysis was then performed to identify patient stratum where the significant HBsAg-HBV DNA correlation was lost. For this subgroup, modulating factors for the DNA-HBsAg relationships were then evaluated by the backward stepwise linear regression method, where the F-test were used to evaluate the model performance. The modulating factors were then introduced into a prediction model. The statistical analysis was performed using the SPSS software (IBM, Armonk, NY). *P* values smaller than 0.05 were considered statistically significant.

## Results

### HBV DNA levels, HBeAg positivity and ISHAK fibrosis stages were independently associated with HBsAg levels

The first cohort comprised 327 chronic hepatitis B patients (Table 1). Age, ISHAK fibrosis stages, HBV DNA levels, hepatitis B e-antigen (HBeAg) positivity, platelet counts and hemoglobin levels were significantly associated with HBsAg levels in the univariate analysis (Table 2). When these variables were entered into multivariate analysis, only three variables remained significantly associated (ISHAK fibrosis stages, HBV DNA levels and HBeAg positivity) (Table 2). Among them, HBV DNA is the most strongly associated variable (*P* < 0.001). An initial model of HBsAg by use of the three independent variables was therefore constructed as a benchmark using the multivariate linear regression as:

**Table 2:** Association of viral and host variables to quantitative HBsAg concentrations using linear regression

	Univariate Analysis			Multivariate Analysis		
Variables	Regression coefficient	95% CI	*P*	Adjusted regression coefficient	95% CI	*P*
Age						
	-0.015	(-0.024 ~ -0.006)	*0.002*	0.002	(-0.009 ~ 0.013)	0.678
Gender-Male						
	-0.188	(-0.491 ~ 0.115)	0.224			
ISHAK Fibrosis Stages						
	-0.134	(-0.206 ~ -0.061)	*<0.001*	-0.125	(-0.203 ~ -0.48)	*0.002*
Piecemeal necrosis						
	-0.098	(-0.223 ~ 0.038)	0.156			
Confluent necrosis						
	-0.073	(-0.210 ~ 0.064)	0.293			
Focal (spotty) lytic necrosis, apoptosis and focal inflammation						
	-0.064	(-0.207 ~ 0.078)	0.375			
Portal inflammation						
	-0.083	(0.197 ~ 0.031)	0.152			
HBV DNA (log10 IU/ml)						
	0.310	(0.258 ~ 0.363)	*<0.001*	0.267	(0.197 ~ 0.337)	*<0.001*
HBeAg						
	0.709	(0.509 ~ 0.909)	*<0.001*	0.301	(0.045 ~ 0.566)	*0.021*
ALT						
	0.000	(0.000 ~ 0.001)	0.23			
AST						
	0.001	(0.000 ~ 0.002)	0.144			
Bilirubin						
	-0.121	(-0.372 ~ 0.130)	0.343			
Albumin						
	-0.017	(-0.455 ~ 0.422)	0.94			
GGT						
	-0.002	(-0.006 ~ 0.001)	0.122			
Platelet						
	0.003	(0.001 ~ 0.005)	*0.003*	0.002	(0.000 ~ 0.005)	0.064
Hemoglobin						
	-0.107	(-0.204 ~ -0.010)	*0.03*	-0.57	(-0.137 ~ 0.023)	0.162

Statistical significant values (*P* <0.05) were now presented in italic font

$$ \mathrm{HBsAg} = 0.274\ *\ \mathrm{H}\mathrm{B}\mathrm{V}\ \mathrm{D}\mathrm{N}\mathrm{A}+0.314\ *\ \left(0\ \mathrm{if}\ \mathrm{HB}\mathrm{eAg}\ \mathrm{negative}\right)\ \hbox{-}\ 0.123\ *\ \mathrm{ISHAK}\ \mathrm{fibrosis}\ \mathrm{score}+1.858 $$

The estimated HBsAg levels by the three-variable model were highly correlated with the measured HBsAg levels (Pearson’s correlation *r* = 0.59; *P* < 0.001). The standard deviation of the regression residual is 0.79 log_10_ IU/ml.

### Identification of a patient subgroup which lacked significant HBV DNA-HBsAg correlations

**Fig. 1:**
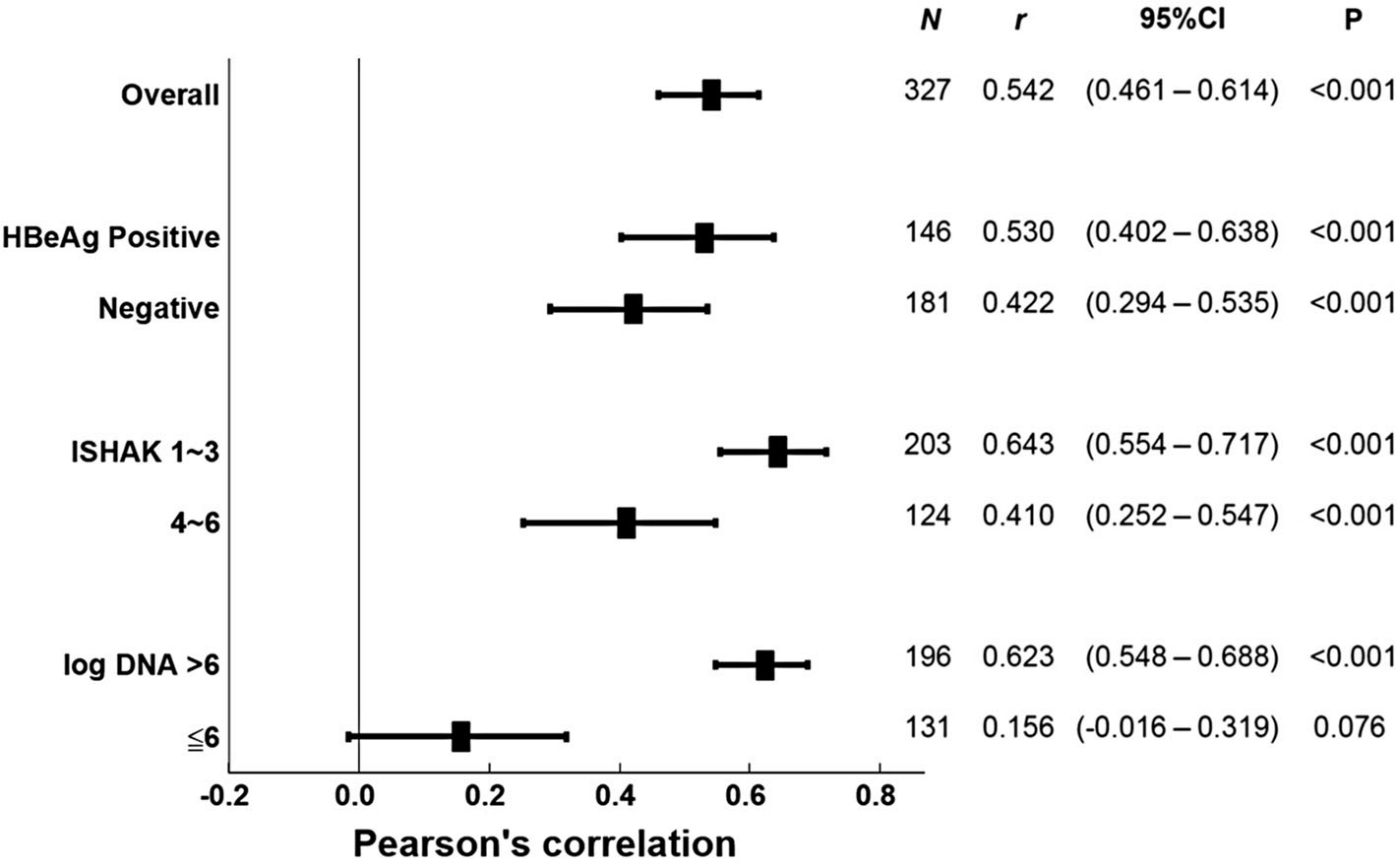
Pearson’s correlation (*r*) of HBsAg and HBV DNA levels in patient subgroups stratified by HBeAg status, ISHAK fibrosis stages and HBV DNA levels

We further conducted the subgroup analysis of patients stratified by the above three variables. Significant HBsAg-DNA correlations remained in HBeAg positive and negative patient subgroups (both *P* < 0.001), in ISHAK score ≥ 4 or ≤ 3 subgroups (both *P* < 0.001), and in patients with HBV-DNA > 6 log_10_ IU/mL (*P* < 0.001). However, no significant association were found in the patient subgroup with HBV-DNA ≤ 6 log_10_ IU/mL (Fig. 1). A baseline comparison of the low- and high- HBV DNA titer subgroups, defined using the boundary threshold of 6 log_10_ IU/mL, showed that the low-titer subgroup has a significantly lower percentage of HBeAg positive patients (27.48%) than the high-titer subgroup (56.12%, Table 3).

**Table 3:** Baseline characteristics of patients with HBV DNA below or above 10^6^ IU/mL

	HBV DNA Low	HBV DNA High	*P*
Subject number	131	196	
Age	45.66 ± 11.62	43.24 ± 10.83	0.059
Gender			
Male	115 (87.79%)	165 (84.18%)	0.363
Female	16 (12.21%)	31 (15.82%)	
Liver Histology			
ISHAK Fibrosis Stages			
0	2 (0.61%)	0 (0%)	0.164
1	15 (11.45%)	25 (12.76%)	
2	16 (12.21%)	35 (17.86%)	
3	53 (40.46%)	57 (29.08%)	
4	11 (8.40%)	22 (11.22%)	
5	29 (22.14%)	45 (22.96%)	
6	5 (3.82%)	12 (6.12%)	
Piecemeal necrosis			
0	45 (34.35%)	56 (28.57%)	0.234
1	68 (51.91%)	97 (49.49%)	
2	15 (11.45%)	32 (16.33%)	
3	3 (2.29%)	11 (5.61%)	
Confluent necrosis			
0	126 (96.18%)	181 (92.35%)	0.368
1	1 (0.76%)	5 (2.55%)	
2	0 (0%)	2 (1.02%)	
3	1 (0.76%)	0 (0%)	
4	3 (2.29%)	7 (3.57%)	
5	0 (0%)	1 (0.51%)	
Focal (spotty) lytic necrosis, /apoptosis and focal inflammation			
0	0 (0%)	2 (1.02%)	0.001
1	53 (40.46%)	40 (20.41%)	
2	54 (41.22%)	107 (54.59%)	
3	21 (16.03%)	46 (23.47%)	
4	3 (2.29%)	1 (0.51%)	
Portal inflammation			
0	4 (3.05%)	2 (1.02%)	0.142
1	38 (29.01%)	39 (19.90%)	
2	36 (27.48%)	67 (34.18%)	
3	49 (37.40%)	74 (37.76%)	
4	4 (3.05%)	13 (6.63%)	
5	0 (0%)	1 (0.51%)	
Viral serology			
HBV DNA (log10 IU/ml)	4.67 ± 1.22	7.48 ± 0.82	<0.001
HBeAg positive	36 (27.48%)	110 (56.12%)	<0.001
HBsAg (log10 IU/ml)	2.83 ± 0.97	3.67 ± 0.83	<0.001
Serum biochemistry			
ALT (IU/L)	161.20 ± 179.34	188.07 ± 168.45	0.175
AST (IU/L)	86.41 ± 89.06	120.77 ± 138.86	0.007
Bilirubin (mg/dL)	1.00 ± 0.50	0.97 ± 0.36	0.559
Albumin (g/dL)	4.58 ± 0.28	4.54 ± 0.39	0.418
Gamma-glutamyltransferase (IU/L)	56.79 ± 42.31	61.58 ± 49.84	0.505
Platelet (1000/mm3)	189.09 ± 49.34	190.23 ± 50.96	0.84
Hemoglobin (g/dL)	15.32 ± 1.34	15.12 ± 1.37	0.26

### A biphasic model of HBsAg concentrations using platelet counts and HBV DNA concentrations

**Fig. 2:**
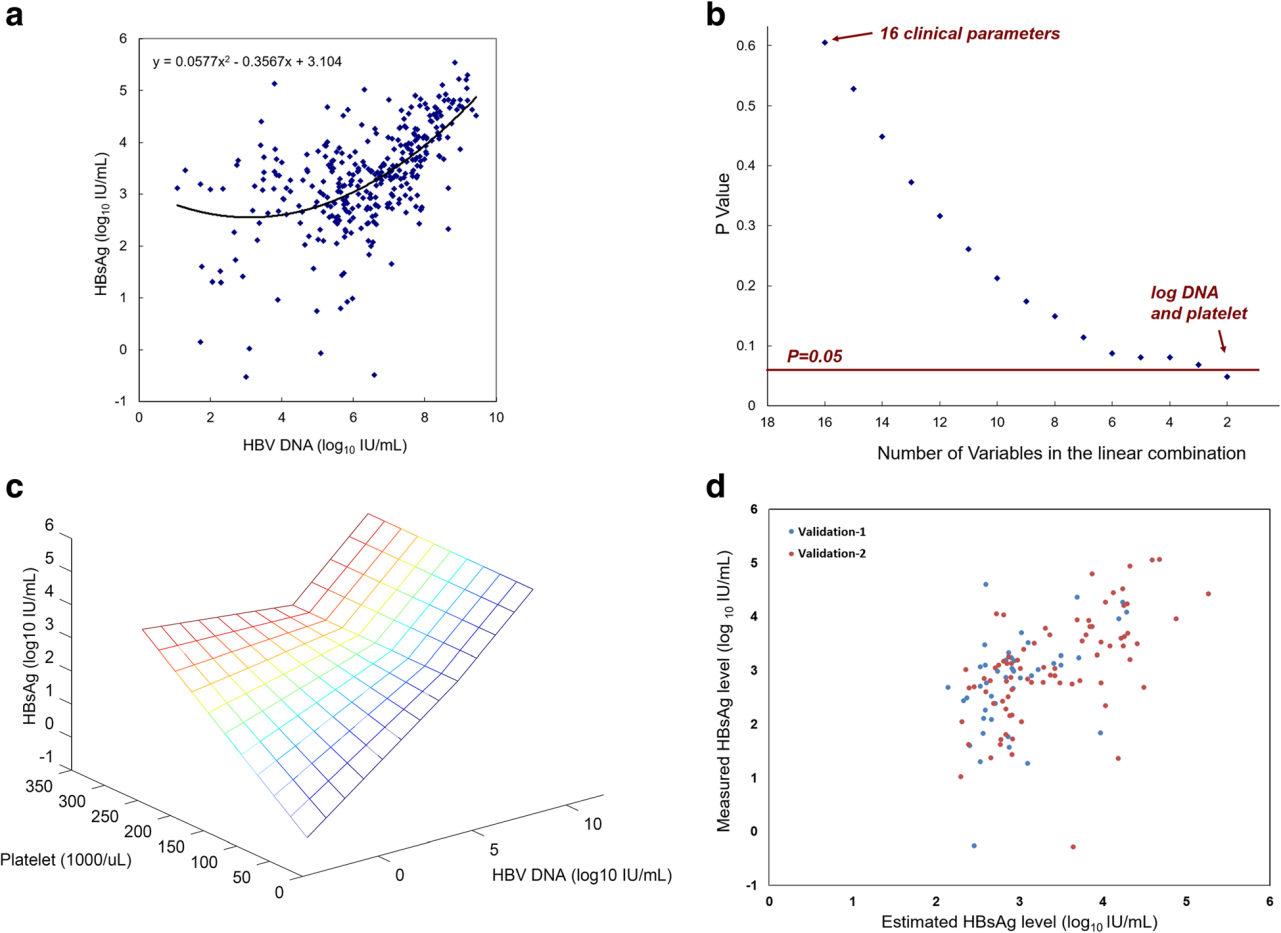
**a** The scatter plot of HBV DNA and HBsAg levels in the model construction cohort. **b** Backward stepwise linear regression analysis in patients with HBV-DNA ≤ 6 log_10_ IU/mL. The x-axis showed the number of variables incorporated in the model, which also equated to the degrees of freedom in the F-test. The y-axis showed the *P* values calculated by the F-test. At the beginning, all 16 clinical variables were incorporated into a linear model. Less relevant variables were progressively removed. At the end of the stepwise process, a linear combination of platelet and HBV DNA levels showed significant association to HBsAg levels (*P* = 0.048, degrees of freedom = 2). **c** Estimated HBsAg levels is a function of HBV DNA levels and platelet counts in the constructed biphasic model. **d** A scatter plot of the measured and estimated HBsAg levels in two validation cohorts. Validation cohort 1: patients with biopsy-included pretreatment evaluations. Validation cohort 2: treatment naïve patients

A scatter plot was then produced to offer a visualization of the relationship between the HBV DNA and HBsAg identified in the previous subgroup analysis (Fig. 2a). Significant HBsAg-DNA correlation were found in patients with HBV-DNA > 6 log_10_ IU/mL but not in patients with HBV-DNA ≤ 6 log_10_ IU/mL, suggesting unknown modulating factors of the HBsAg levels in the HBV DNA low-titer subgroup. Therefore, a backward stepwise linear regression analysis was then performed in the subgroup when HBV-DNA ≤ 6 log_10_ IU/mL (*N* = 131). This was done by incorporating all the 16 clinical variables into a multivariate linear regression equation, then gradually removing irrelevant variables one at a time, and evaluating the statistical significance (Fig. 2b). At the end of the stepwise analysis, the linear combination of two variables, platelet counts and DNA levels, was found to be significantly correlated with HBsAg levels (F-test *P* = 0.048, degrees of freedom = 2).

We continued to construct a biphasic model of HBsAg level using (i) HBV-DNA alone when HBV-DNA > 6 log_10_ IU/mL, and (ii) HBV-DNA and platelet counts together when HBV-DNA ≤ 6 log_10_ IU/mL.

$$ \mathrm{HB}\mathrm{sAg}=0.538\ast \mathrm{H}\mathrm{B}\mathrm{V}\mathrm{D}\mathrm{N}\mathrm{A}+0.001\ast \mathrm{platelet}\ast \left(\left|6{\textstyle \hbox{-}}\mathrm{HBVDNA}\right|+6{\textstyle \hbox{-}}\mathrm{HBVDNA}\right){\textstyle \hbox{-} }0.321 $$

Where |‧| represented the absolute-value function. The relationship between the HBV DNA levels, platelet counts and the estimated HBsAg levels was visualized in Fig. 2c. In the model construction cohort, the HBsAg levels calculated by the biphasic model were significantly correlated with the measured HBsAg levels (*r* = 0.60, *P* < 0.001). The standard deviation of the regression residual is 0.78 log_10_ IU/ml.

Clinical records of additional 45 patients with liver biopsy-included pretreatment evaluations were used for the first validation (Table 4)**.** Significant positive correlations were found between estimated and measured HBsAg concentrations (*r* = 0.47, *P* = 0.001). The standard deviation of the residual is 0.82 log_10_ IU/mL. Furthermore, a cohort of 80 treatment-naïve patients (not receiving pretreatment liver biopsy) evaluated between 2010-2012 were recruited for the second validation (Table 5)**.** Significant positive correlations were found again (*r* = 0.57, *P* < 0.001). The standard deviation of the residual is 0.88 log_10_ IU/mL. A visual presentation of the estimated and the measured HBsAg levels in the two validation cohorts were shown in Fig. 2d.

**Table 4:** Baseline characteristics of patients in the first validation cohort. The patients all received biopsy-included pretreatment evaluations between 2007-2009

	Values	*P* ^a^
Subject number	45	
Age	51.53 ± 10.30	<0.001
Gender		0.034
Male	33 (73.33%)	
Female	12 (26.67%)	
Liver Histology		
ISHAK Fibrosis Stages		<0.001
1	7 (15.56%)	
2	5 (11.11%)	
3	13 (28.89%)	
4	6 (13.33%)	
5	10 (22.22%)	
6	14 (31.11%)	
Piecemeal necrosis		0.07
0	10 (22.22%)	
1	23 (51.11%)	
2	6 (13.33%)	
3	6 (13.33%)	
Confluent necrosis		0.165
0	40 (88.89%)	
4	5 (11.11%)	
Focal (spotty) lytic necrosis, apoptosis and focal inflammation		0.329
1	14 (31.11%)	
2	27 (60.00%)	
3	4 (8.89%)	
Portal inflammation		0.114
0	2 (4.44%)	
1	13 (28.89%)	
2	6 (13.33%)	
3	23 (51.11%)	
4	1 (2.22%)	
Viral serology		
HBV DNA (log10 IU/ml)	5.31 ± 1.68	<0.001
HBsAg (log10 IU/ml)	2.79 ± 0.90	<0.001
Hematology		
ALT (IU/L)	141.00 ± 124.94	0.087
AST (IU/L)	91.29 ± 83.45	0.277
Bilirubin (mg/dL)	0.90 ± 0.28	0.119
Albumin (g/dL)	4.45 ± 0.28	0.196
Gamma-glutamyltransferase (IU/L)	66.20 ± 65.26	0.626
Platelet (1000/mm3)	179.36 ± 53.17	0.22
Hemoglobin (g/dL)	14.69 ± 1.29	0.032

^a^ Compared with the model-construction cohort

**Table 5:** Baseline characteristics of patients in the second validation cohort. The patients were treatment-naïve patients evaluated between 2010-2012

	Values	*P* ^a^
Subject number	80	
Age	49.01 ± 11.69	0.001
Gender		0.002
Male	57 (71.25%)	
Female	23 (28.75%)	
HBV DNA (log_10_ IU/ml)	6.57 ± 1.78	0.339
HBeAg positive	27 (33.75%)	0.077
HBsAg (log_10_ IU/ml)	3.05 ± 0.97	0.022
Platelet (1000/mm3)	166.61 ± 64.06	0.003

^a^ Compared with the model-construction cohort

## Discussion

Chronic hepatitis B often lasted for decades, if not lifetime. The HBsAg level was high in the immune tolerance phase. It reduced gradually in the immune clearance phase and the inactive residual phases [12]. A strong positive linear relationship between age and the annual rate of HBsAg seroclearance has been demonstrated in a meta-analysis of 13 study cohorts [26]. The highest rate of HBsAg seroclearance occurred at 50 years old [26], an age when many patients have already developed mild or severe liver fibrosis. The negative correlations between fibrosis stages and HBsAg levels has also been demonstrated in previous univariate analyses [27, 28]. Significantly lower HBsAg levels were found in patients with ISHAK fibrosis score >1, compared with those with score ≤ 1 (*P* < 0.001) [27]. Baseline data from a multicenter, phase III trial of peginterferon alfa-2a and a phase IV NEPTUNE trial showed that lower HBsAg levels were associated with lower PS1 and PS2 scores, which indicated more severe fibrosis [28].

The necessity of multivariate analysis arises as multiple factors (age, HBsAg level, fibrosis stage) were shown to be involved in univariate analyses [27, 28]. Our systematical evaluation of hematological, histological and viral serological variables showed that the progression of liver fibrosis was accompanied by HBsAg reduction (Table 2 **,** adjusted regression coefficient of “ISHAK fibrosis stage” = -0.125, *P* = 0.002), independent of age, HBV DNA levels, HBeAg positivity, platelet counts and hemoglobin levels. Age on the other hand was negatively correlated with HBsAg concentrations only in the univariate analysis but not in the multivariate analysis.

The discrepancy between HBV DNA and HBsAg levels underlies the reason why HBsAg cannot play comparable roles on the estimation of subsequent HCC risks as what HBV DNA can do (except for patients with very low levels of HBV DNA). HBV DNA has been established as an important predictor of HCC risks [29]. A recent report showed that HBV DNA in general is a better predictor of HCC than HBsAg [13]. However, in a specific subgroup of HBeAg negative, HBV DNA < 2000 IU/mL patients, HBsAg rather than HBV DNA was a better predictor [13]. This conclusion was based on a study population of non-cirrhotic, relatively young patients (>50% patients were 28–39 years old at the time of enrolment). Considering the strong effect of fibrosis stages on the subsequent HCC occurrence [12, 30] and the negative correlations between HBsAg levels and fibrosis stages demonstrated here, it was reasonable to say that any potential positive correlations between HBsAg and HCC incidence can only be found in patients with similar fibrosis status, which however required liver biopsy to be assessed correctly. The predictive role of HBsAg on HCC reported in [13] may not be readily extrapolated to elder people with mild, moderate and severe fibrosis.

A model of HBsAg levels can be constructed straightforwardly using the three independent variables (HBV DNA, fibrosis stages and HBeAg status). This model was a benchmark in the search for a simpler model with fewer number of clinical variables. We continued to investigate patient subgroups stratified by the three independent variables. We found that the DNA remained significantly associated with HBsAg in all strata except when DNA < 6 log_10_ IU/mL. A backward stepwise linear regression analysis in the low-titer subgroup showed that, after the less relevant variables were removed gradually, platelet counts and HBV DNA remained, and their combination was synergistically associated with HBsAg levels. Thus, a biphasic model was constructed using HBV DNA alone when HBV-DNA > 6 log_10_ IU/mL, and platelet levels in conjunction with HBV DNA when HBV-DNA ≤ 6 log_10_ IU/mL. This new model is simpler, with fewer variables, yet the correlation (*r* = 0.60) is even higher and the standard deviation of the regression residual (*e* = 0.78 IU/mL) is even lower than those of the three-variable model (*r* = 0.59 and e = 0.79 IU/mL).

The reduction of platelet counts, i.e. thrombocytopenia, has been acknowledged to be associated with chronic liver diseases and cirrhosis [31, 32]. The correlation between platelet counts and ISHAK fibrosis stages made them both associated with HBsAg levels in our univariate analysis (Table 2). When they were both introduced into the multivariate analysis, only the ISHAK stage but not the platelet counts (*P* = 0.064) remained significantly associated. However, in the low-titer subgroup when HBV-DNA ≤ 6 log_10_ IU/mL, platelet counts rather than ISHAK stages were remained in the backward stepwise regression analysis. This showed that platelet counts and HBV DNA formed an effective combination for estimating HBsAg when HBV-DNA ≤ 6 log_10_ IU/mL.

Platelets were widely known for their roles in blood coagulation. In addition to this conventional role, its antimicrobial roles were gradually being noticed [33]. Platelets can secret chemokine ligand 5 (CCL5) so as to stimulate the production of megakaryocytes, forming a positive feedback loop of platelet activation [34]. It can also secrete hepatocyte growth factor (HGF) so as to protect against liver fibrosis [35]. The detailed mechanism on the interactions of platelets to the HBV life cycle warrants further investigations.

The quantitative modeling provided a numerical basis for our understanding on the relationship between HBsAg, HBV DNA, age, fibrosis stages and platelet counts. The estimated HBsAg concentrations correlated well with the measured HBsAg in the model construction cohort as well as the two independent validation cohorts (*P* ≤ 0.001 in all), supporting the use of the biphasic model in retrospective studies where the HBsAg was not measured at previous timepoints and no stored clinical samples were available. Since quantitative HBsAg measurement has become more and more available recently, patients’ HBsAg levels can now be measured directly without the help of this biphasic model.

Patients in the immune activation and the inactive residual phases were particularly required for quantitative HBsAg monitoring, and they were the major population of our study cohorts. Although we have analyzed a total of 452 patients, patients in the immune tolerance phases were not well represented. Therefore, the current analysis may only be applied to patients in the immune activation phase onward, but may not be extrapolated to patients in the immune tolerance phase.

In conclusion, serum HBsAg levels depended on HBV DNA titers, the liver fibrosis stages, and HBeAg positivity. Taking into consideration of all the above aspects, we constructed a noninvasive, biphasic quantitative model using two variables, HBV DNA and platelet levels, which can effectively estimate HBsAg concentrations.

## Abbreviations

ALT: Alanine aminotransferase
AST: Aspartate aminotransferase
DNA: Deoxyribonucleic acid
HBeAg: Hepatitis B e-antigen
HBsAg: Hepatitis B surface antigen
HBV: Hepatitis B virus
HCC: Hepatocellular carcinoma

## References

[1] Lavanchy D (2004). Hepatitis B virus epidemiology, disease burden, treatment, and current and emerging prevention and control measures. J Viral Hepat.

[2] Liaw Y-F, Chu C-M (2009). Hepatitis B virus infection. Lancet.

[3] Elgouhari HM, Abu-Rajab Tamimi TI, Carey WD (2008). Hepatitis B virus infection: Understanding its epidemiology, course, and diagnosis. Cleve Clin J Med.

[4] Yim HJ, Lok AS-F (2006). Natural history of chronic hepatitis B virus infection: What we knew in 1981 and what we know in 2005. Hepatology.

[5] Ganem D, Prince AM (2004). Hepatitis B virus infection — natural history and clinical consequences. N Engl J Med.

[6] Pan CQ: Natural History and Clinical Consequences of Hepatitis B Virus Infection. International Journal of Medical Sciences 2005:36. 10.7150/ijms.2.36 PMC1142223 15968338

[7] Poordad F (2007). Review article: thrombocytopenia in chronic liver disease. Aliment Pharmacol Ther.

[8] Bernardi M, Maggioli C, Zaccherini G (2012). Human Albumin in the Management of Complications of Liver Cirrhosis. Crit Care.

[9] Garcia–Tsao G (2001). Current management of the complications of cirrhosis and portal hypertension: variceal hemorrhage, Ascites, and spontaneous bacterial peritonitis. Gastroenterology.

[10] Liang KH, Hsu CW, Chang ML, Chen YC, Lai MW, Yeh CT (2016). Peginterferon is superior to nucleos(t)ide analogs for prevention of hepatocellular carcinoma in chronic hepatitis B. J Infect Dis.

[11] Lok ASF, McMahon BJ (2009). Chronic hepatitis B: update 2009. Hepatology.

[12] Liaw Y-F, Kao J-H, Piratvisuth T, Chan HLY, Chien R-N, Liu C-J, Gane E, Locarnini S, Lim S-G, Han K-H (2012). Asian-Pacific consensus statement on the management of chronic hepatitis B: a 2012 update. Hepatol Int.

[13] Tseng TC, Liu Cj Fau - Yang H-C, Yang Hc Fau - Su T-H, Su Th Fau - Wang C-C, Wang Cc Fau - Chen C-L, Chen Cl Fau - Kuo SF-T, Kuo Sf Fau - Liu C-H, Liu Ch Fau - Chen P-J, Chen Pj Fau - Chen D-S, Chen Ds Fau - Kao J-H, et al. High levels of hepatitis B surface antigen increase risk of hepatocellular carcinoma in patients with low HBV load. 2012;142(5):1140-49. 10.1053/j.gastro.2012.02.007 22333950

[14] Clinical Practice Guidelines EASL (2012). Management of chronic hepatitis B virus infection. J Hepatol.

[15] Liaw Y-F, Sung JJY, Chow WC, Farrell G, Lee C-Z, Yuen H, Tanwandee T, Tao Q-M, Shue K, Keene ON (2004). Lamivudine for patients with chronic hepatitis B and advanced liver disease. N Engl J Med.

[16] Marcellin P, Heathcote EJ, Buti M, Gane E, de Man RA, Krastev Z, Germanidis G, Lee SS, Flisiak R, Kaita K (2008). Tenofovir disoproxil fumarate versus Adefovir dipivoxil for chronic hepatitis B. N Engl J Med.

[17] Chang T-T, Gish RG, de Man R, Gadano A, Sollano J, Chao Y-C, Lok AS, Han K-H, Goodman Z, Zhu J (2006). A comparison of entecavir and lamivudine for HBeAg-positive chronic hepatitis B. N Engl J Med.

[18] Liaw YF, Gane E, Leung N, Zeuzem S, Wang Y, Lai CL, Heathcote EJ, Manns M, Bzowej N, Niu J (2009). 2-year GLOBE trial results: telbivudine is superior to lamivudine in patients with chronic hepatitis B. Gastroenterology.

[19] Pol S, Lampertico P (2012). First-line treatment of chronic hepatitis B with entecavir or tenofovir in ‘real-life’ settings: from clinical trials to clinical practice. J Viral Hepat.

[20] Ocana S (2011). Diagnostic strategy for occult hepatitis B virus infection. World J Gastroenterol.

[21] Said ZNA (2011). An overview of occult hepatitis B virus infection. World J Gastroenterol.

[22] Zobeiri M (2013). Occult hepatitis B: clinical viewpoint and management. Hepat Res Treat.

[23] Chan HL-Y, Thompson A, Martinot-Peignoux M, Piratvisuth T, Cornberg M, Brunetto MR, Tillmann HL, Kao J-H, Jia J-D, Wedemeyer H (2011). Hepatitis B surface antigen quantification: why and how to use it in 2011 – a core group report. J Hepatol.

[24] Liaw Y-F (2011). Clinical utility of hepatitis B surface antigen quantitation in patients with chronic hepatitis B: A review. Hepatology.

[25] Ishak K, Baptista A, Bianchi L, Callea F, De Groote J, Gudat F, Denk H, Desmet V, Korb G, MacSween RNM (1995). Histological grading and staging of chronic hepatitis. J Hepatol.

[26] Chu C-M, Liaw Y-F (2010). Hepatitis B surface antigen seroclearance during chronic HBV infection. Antivir Ther.

[27] Seto W-K, Wong DK-H, Fung J, Ip PPC, Yuen JC-H, Hung IF-N, Lai C-L, Yuen M-F (2012). High hepatitis B surface antigen levels predict insignificant fibrosis in hepatitis B e antigen positive chronic hepatitis B. PLoS One.

[28] Marcellin P, Martinot-Peignoux M, Asselah T, Batrla R, Messinger D, Rothe V, Lau G, Liaw Y-F (2015). Serum levels of hepatitis B surface antigen predict severity of fibrosis in patients with E antigen-positive chronic hepatitis B. Clin Gastroenterol Hepatol.

[29] Yang H-I, Yuen M-F, Chan HL-Y, Han K-H, Chen P-J, Kim D-Y, Ahn S-H, Chen C-J, Wong VW-S, Seto W-K (2011). Risk estimation for hepatocellular carcinoma in chronic hepatitis B (REACH-B): development and validation of a predictive score. Lancet Oncol.

[30] Bruix J, Sherman M (2011). Management of hepatocellular carcinoma: an update.

[31] Vallet-Pichard A, Mallet V, Nalpas B, Verkarre V, Nalpas A, Dhalluin-Venier V, Fontaine H, Pol S (2007). FIB-4: an inexpensive and accurate marker of fibrosis in HCV infection. Comparison with liver biopsy and fibrotest. Hepatology.

[32] Wai C (2003). A simple noninvasive index can predict both significant fibrosis and cirrhosis in patients with chronic hepatitis C. Hepatology.

[33] Yeaman MR (2014). Platelets: at the nexus of antimicrobial defence. Nat Rev Microbiol.

[34] Machlus KR, Johnson KE, Kulenthirarajan R, Forward JA, Tippy MD, Soussou TS, El-Husayni SH, Wu SK, Wang S, Watnick RS (2015). CCL5 derived from platelets increases megakaryocyte proplatelet formation. Blood.

[35] Kodama T, Takehara T, Hikita H, Shimizu S, Li W, Miyagi T, Hosui A, Tatsumi T, Ishida H, Tadokoro S (2010). Thrombocytopenia exacerbates cholestasis-induced liver fibrosis in mice. Gastroenterology.

